# *In vitro* antifungal activity of biosynthesized selenium nanoparticles using plant extracts and six comparators against clinical *Fusarium* strains

**DOI:** 10.22034/CMM.2024.345189.1504

**Published:** 2023-12

**Authors:** Mohsen Nosratabadi, Mohammad Ali Ebrahimzadeh, Seyedeh Roya Alizadeh, Iman Haghani, Leila Faeli, Robab Ebrahimi Barogh, Abdullah M.S. Al Hatmi, Mahdi Abastabar

**Affiliations:** 1 Student Research Committee, Mazandaran University of Medical Sciences, Sari, Iran; 2 Department of Medical Mycology, School of Medicine, Mazandaran University of Medical Sciences, Sari, Iran; 3 Invasive Fungi Research Center, Communicable Diseases Institute, Mazandaran University of Medical Sciences, Sari, Iran; 4 Department of Medicinal Chemistry, School of Pharmacy and Pharmaceutical Sciences Research Center, Mazandaran University of Medical Sciences, Sari, Iran; 5 Natural and Medical Sciences Research Center, University of Nizwa, Nizwa, Oman

**Keywords:** Antifungal activity, *Fusarium* species, Se NPs, Plant extract

## Abstract

**Background and Purpose:**

*Fusarium* species are commonly resistant to many antifungal drugs. The limited therapeutic options available have led to a surge of research efforts aimed at discovering novel antifungal compounds in recent decades. This study aimed to assess the *in vitro* antifungal activity of plant-based biosynthesized selenium nanoparticles (Se NPs) and six comparators against a set of clinical *Fusarium* strains.

**Materials and Methods:**

*In vitro* antifungal activity of Se NPs synthesized using plant extracts of *Allium paradoxum*, *Crocus caspius*, *Pistacia vera* L. hull, *Vicia faba* L. hull and *Heracleum persicum*, as well as six common antifungal drugs, namely voriconazole, itraconazole, amphotericin B, posaconazole, natamycin, and caspofungin were evaluated against 94 clinical *Fusarium* strains using broth microdilution according to Clinical and Laboratory Standards Institute guideline.

**Results:**

The obtained results were intriguing since all five types of biosynthesized Se NPs demonstrated significantly higher antifungal activity, compared to antifungal drugs. It was found that Se NPs synthesized by *V. faba* L. hull extract (0.03 μg/ml) had the lowest geometric mean minimum inhibitory concentration value followed by Se NPs synthesized by *P. vera* L. hull extract (0.25 μg/ml), *A. paradoxum* extract (0.39 μg/ml), *C. caspius* extract (0.55 μg/ml), and *H. persicum* extract (0.9 μg/ml).

**Conclusion:**

Plant-based Se NPs demonstrated supreme antifungal activity and could be considered promising antifungal agents for *Fusarium* infections. However, tests, such as toxicity and *in vivo* tests are needed before the product can be used in clinical settings.

## Introduction

Fungal pathogens pose a serious threat to public health, affecting at least 1.5 million people worldwide each year [ 1 , 2 ]. Resistance to conventional antifungal drugs has emerged as a global public health concern, severely limiting available treatment options [ 3 - 6 ]. Furthermore, since fungal and human cells are so similar, it is difficult to discover and develop new and effective antifungal drugs [ 7 , 8 ].

*Fusarium* is a large and remarkably diverse genus of saprophytic fungi found throughout nature [ 9 ]. *Fusarium* species are important phytopathogenic and mycotoxin-producing fungi [ 10 , 11 ]. They are also opportunistic fungi that cause a wide range of infections, from superficial and localized infections, such as onychomycosis and keratitis in healthy people to fatal systemic infections in severely ill people [ 12 - 14 ].

 In the United States and Europe, *Fusarium* is the second most common mold-causing human infection [ 15 - 17 ]. This fungal genus has high levels of intrinsic resistance to commonly available antifungal agents, posing a serious challenge to healthcare systems all over the world [ 15 , 17 ]. Since *Fusarium* species are frequently resistant to many antifungal drugs, and since there is a limited therapeutic repository for these fungal infections, research focused on finding novel and potent antifungal compounds as well as alternatives are urgently needed [ 12 , 18 - 20 ].

 Development of nanotechnology-based therapies in recent years has opened up new avenues for the treatment of drug-resistant fungal infections. Due to their nanometer size and large surface areas, nanoparticles have unique physicochemical properties that increase interactions with microbial cells and significantly influence their antimicrobial effects [ 21 , 22 ]. 

Selenium is a trace element that is essential for human and animal health, and it is involved in antioxidant defense, metabolism, and detoxification. Due to their low toxicity, high biodegradability, and bioavailability as well as anticancer, antidiabetic, antioxidant, antibacterial, antiprotozoal, antiviral, antifungal, and antibiofilm properties, selenium nanoparticles (Se NPs) are among the most appealing nanomaterials in biomedicine [ 23 , 24 ]. 

According to some studies, the antimicrobial activity of Se NPs is often attributed to the generation of reactive oxygen species, which can damage the DNA and cell membrane. However, the precise antimicrobial mechanisms of Se NPs have not been fully elucidated, and more research is needed. According to previous research, Se NPs can be synthesized through chemical, physical, or biological processes [ 25 - 28 ]. However, green synthesis or biologically synthesized Se NPs using microorganisms or plant extracts as natural reducing agents provides novel, simple, low-cost, non-toxic, and environment-friendly methods for the production of Se NPs [ 29 - 32 ]. 

Some plants contain a high concentration of phytochemical compounds with important biological and medicinal properties. The efficiency and capability of these compounds as nanoparticle reductants in green synthesis have previously been demonstrated [ 31 - 33 ]. The current study aimed to assess the *in vitro* antifungal activity of Se NPs made from extracts of *Allium paradoxum*, *Crocus caspius*, *Pistacia vera* L. hull, *Vicia faba* L. hull, and *Heracleum persicum*, as well as six antifungal drugs, namely, voriconazole, itraconazole, amphotericin B, posaconazole, natamycin, and caspofungin against a collection of clinical *Fusarium* strains.

## Materials and Methods

### Strains

In this study, 94 clinical isolates of *Fusarium* were examined. Isolates were collected from different medical mycology centers in Iran between 2019 and 2022 [ 34 ]. The most isolates were recovered from nail samples (58.51%, 55/94), followed by cornea (40.43%, 38/94) and Sinus (1.06%, 1/94). Partial sequencing of the translation elongation factor 1-alpha (*TEF-1α*) identified all *Fusarium* isolates at the species level.

### Synthesis of plant-based selenium nanoparticles

The plant-based Se NPs used in this study were synthesized by the Department of Medicinal Chemistry at the School of Pharmacy and Pharmaceutical Sciences Research Centre of Mazandaran University of Medical Sciences, Sari, Iran [ 31 , 32 ]. The plants *A. paradoxum*, *C. caspius*, *P. vera* L. hull, V. faba L. hull, and *H. persicum* were dried in daylight, cut into small pieces (2-3 mm), and stored at room temperature. Each plant (10 g) was combined with 80 mL of deionized water and heated at 50 °C for 1 h. The mixture was then sonicated for 30 min and filtered through the Whatman filter paper. In the process of Se NPs biosynthesis using plant extracts, 17.3 mg of Na_2_SeO_3_ was dissolved in 10 mL of deionized water and stirred at 45-50 °C and 500 rpm. Subsequently, 5 mL of the aqueous extract was added to the reaction mixture drop by drop. After two days, the color of the reaction changed from colorless to reddish, indicating the reduction of Se ions.

### Antifungal susceptibility testing

The *in vitro* antifungal activities of biosynthesized Se NPs using plant extracts of *A. paradoxum* (A-Se NPs), *C. caspius* (C-Se NPs), *P. vera* L. hull (P-Se NPs), *V. faba* L. hull (V-Se NPs), and *H. persicum* (H-Se NPs) as well as six routine antifungal drugs, including itraconazole (Janssen, Beerse, Belgium), posaconazole (Pfizer, Sandwich, United Kingdom), voriconazole (Pfizer, Sandwich, United Kingdom), natamycin (Sigma-Aldrich, Steinheim, Germany), amphotericin B (Bristol-Myers-Squib, Woerden, The Netherlands), and caspofungin (Merck Sharp & Dohme BV) were determined against 94 clinical *Fusarium* isolates. It was performed by using broth microdilution according to the Clinical and Laboratory Standards Institute M38-A3 [ 35 ]. 

The final concentrations of agents in the wells ranged from 0.016 to 16 μg/ml for V-Se NPs, A-Se NPs, P-Se NPs, C-Se NPs, H-Se NPs, amphotericin B, voriconazole, natamycin, posaconazole, itraconazole, and 0.008 to 8 μg/ml for caspofungin. 

The *Fusarium* isolates were cultured on Sabouraud dextrose agar (Difco Laboratories, Detroit, MI, USA) and incubated at 35 °C for 5 to 7 days for adequate sporulation. Suspensions were diluted at 1:50 in RPMI 1640 medium to obtain final inoculum between 0.4 × 10^4^ to 5 × 10^4^ CFU/ml. Plates were incubated at 35 °C, and minimum inhibitory concentrations (MICs) and minimum effective concentrations (MECs) were read after 48 h. 

The MIC was determined visually as the lowest concentration of agent that resulted in 100% inhibition of fungal growth while for caspofungin MEC was microscopically determined as the lowest concentration of drug that resulted in the growth of compact hyphal forms compared with growth control. *Candida krusei* (ATCC 6258), *Candida parapsilosis* (ATCC 22019), and *Aspergillus flavus* (ATCC 2004304) served as quality control strains.

## Results

All *Fusarium* strains were previously identified using *TEF1* partial gene analysis [ 34 ].

The species used in the study belonged to the *Fusarium solani* species complex (n=52), *F. fujikuroi* species complex (n=33), *F. oxysporum* species complex (n=7), *F. incarnatum* equiseti species complex (n=1), and F. sambucinum species complex (n=1) (Table 1). The geometric mean (GM) MICs/MECs, MIC/MEC ranges, MIC_50_/MEC_50_, and MIC_90_/MEC_90_ distributions of the tested compounds are shown in Table 2 and Table 3.

**Table 1:** Sources and the number of the *Fusarium* isolates based on the species complexes

*Fusarium* complexes	Species	Source	No.
***F. fujikuroi* species complex (n=33)**	*F. proliferatum*	Nail	15
		Cornea	6
		Sinus	1
	*F. fujikuroi*	Nail	3
	*F. acutatum*	Nail	1
	*F. verticillioides*	Nail	5
	*F. thapsinum*	Cornea	2
***F. solani* species complex (n=52)**	*F. falciforme*	Nail	2
		Cornea	3
	*F. keratoplasticum*	Nail	6
	*F. solani* sensu stricto	Cornea	22
		Nail	19
***F. sambucinum* species complex (n=1)**	*F. brachygibbosum*	Cornea	1
***F. incarnatum* equiseti species complex (n=1)**	*F. equiseti*	Nail	1
***F. oxysporum* species complex (n=7)**	*F. oxysporum*	Cornea	4
		Nail	3

**Table 2:** In vitro susceptibilities of plant-based selenium nanoparticles in comparison with six antifungal drugs against 94 clinical *Fusarium* isolates

*Fusarium* species	Evaluated compounds	MIC_50_ (μg/ml)	MIC_90_ (μg/ml)	MIC range (μg/ml)	GM (μg/ml)	Mode (μg/ml)
Total *Fusarium* isolates (n=94)	A-Se NPs	0.5	2	0.016-8	0.39	1
	C-Se NPs	0.5	4	0.016-8	0.55	0.5
	P-Se NPs	0.25	4	0.016-16	0.25	0.5
	V-Se NPs	0.032	0.125	0.016-0.25	0.03	0.016
	H-Se NPs	1	4	0.016-8	0.9	1
	VRC	4	8	0.25-16	3.25	4
	ITC	16	16	0.032-16	13.01	16
	AMB	1	4	0.125-16	1.05	1
	NAT	4	8	0.125-16	3.91	8
	CAS	8	8	0.064-8	7	8
	POS	8	16	0.25-16	5.29	8
*F. solani* complex (n=52)	A-Se NPs	1	4	0.016-8	0.56	1
	C-Se NPs	1	4	0.016-8	0.64	2
	P-Se NPs	0.5	8	0.016-16	0.46	0.5
	V-Se NPs	0.032	0.125	0.016-0.25	0.03	0.016
	H-Se NPs	1	8	0.064-8	1.32	1
	VRC	4	8	0.25-16	3.74	8
	ITC	16	16	0.032-16	11.93	16
	AMB	0.5	2	0.125-16	0.67	0.5
	NAT	8	8	0.5-16	5.15	8
	CAS	8	8	0.064-8	6.46	8
	POS	8	16	0.5-16	6.55	16
*F. fujikuroi* complex (n=33)	A-Se NPs	0.25	1	0.016-4	0.23	0.5
	C-Se NPs	0.5	2	0.016-4	0.48	0.5
	P-Se NPs	0.064	1	0.016-4	0.1	0.016
	V-Se NPs	0.032	0.125	2-16	0.03	0.016
	H-Se NPs	0.5	4	0.064-8	0.56	0.25
	VRC	4	8	1-8	2.74	4
	ITC	16	16	8-16	14.1	16
	AMB	2	8	0.125-16	1.72	1
	NAT	4	8	0.125-8	2.41	4
	CAS	8	8	4-8	7.67	8
	POS	4	16	0.25-16	4.17	16
*F. oxysporum* complex (n=7)	A- Se NPs	-	-	0.125-2	0.37	0.125
	C- Se NPs	-	-	0.5-1	0.61	0.5
	P- Se NPs	-	-	0.125-0.5	0.18	0.125
	V- Se NPs	-	-	0.016-0.064	0.03	0.064
	H- Se NPs	-	-	0.5-2	0.6	0.5
	VRC	-	-	1-8	2.43	2
	ITC	-	-	16	16	16
	AMB	-	-	0.5-16	2.69	2
	NAT	-	-	2-8	4	8
	CAS	-	-	8	8	8
	POS	-	-	0.5-16	4.41	8
*F. incarnatum* equiseti species complex (n=1)	A-Se NPs	-	-	0.25	-	-
	C-Se NPs	-	-	0.5	-	-
	P-Se NPs	-	-	1	-	-
	V-Se NPs	-	-	0.016	-	-
	H-Se NPs	-	-	2	-	-
	VRC	-	-	4	-	-
	ITC	-	-	16	-	-
	AMB	-	-	1	-	-
	NAT	-	-	8	-	-
	CAS	-	-	8	-	-
	POS	-	-	8	-	-
*F. sambucinum* species complex (n=1)	A-Se NPs	-	-	0.125	-	-
	C-Se NPs	-	-	0.016	-	-
	P-Se NPs	-	-	0.125	-	-
	V-Se NPs	-	-	0.016	-	-
	H-Se NPs	-	-	0.016	-	-
	VRC	-	-	4	-	-
	ITC	-	-	16	-	-
	AMB	-	-	1	-	-
	NAT	-	-	8	-	-
	CAS	-	-	8	-	-
	POS	-	-	2	-	-

MIC: minimum inhibitory concentration, GM: geometric mean, A-Se NPs: *A. paradoxum*-selenium nanoparticles, C-Se NPs: *C. caspius*-selenium nanoparticles, P-Se NPs: *P. vera* L. hull-selenium nanoparticles, V-Se NPs: *V. faba* L. hull-selenium nanoparticles, H-Se NPs: *H. persicum*-selenium nanoparticles, VRC: voriconazole, ITC: itraconazole, AMB: amphotericin B, NAT: natamycin, CAS: caspofungin, POS: posaconazole

**Table 3:** Minimum inhibitory concentrations of five Se NPs and six antifungal drugs on 94 clinical *Fusarium* isolates

Fusarium species	Evaluated compounds	MIC distribution (μg/ml)										
		0.016	0.032	0.064	0.125	0.25	0.5	1	2	4	8	16
Total *Fusarium* isolates (n=94)	A-Se NPs	6	2	3	16	15	17	21	6	4	4	
	C-Se NPs	6	3	2	10	10	20	12	16	12	3	
	P-Se NPs	15	5	7	18	7	18	6	2	9	5	2
	V-Se NPs	43	19	20	11	1						
	H-Se NPs	1		2	6	12	18	21	15	12	7	
	VRC					2	3	13	14	34	27	1
	ITC		1			1				1	11	80
	AMB				6	5	23	30	14	9	2	5
	NAT				1	1	5	8	11	22	44	2
	CAS			1					1	9	83	
	POS					2	6	2	14	15	28	27
F. solani complex (n=52)	A-Se NPs	3	1	1	5	9	6	15	5	3	4	
	C-Se NPs	4	2	1	6	5	5	6	11	9	3	
	P-Se NPs	6	3	1	8	2	13	4	1	7	5	2
	V-Se NPs	22	11	11	7	1						
	H-Se NPs			1	1	4	10	11	10	9	6	
	VRC					2	3	2	7	16	21	1
	ITC		1			1				1	5	44
	AMB				5	4	19	17	4	1	1	1
	NAT						4	1	2	12	31	2
	CAS			1					1	7	43	
	POS						2		8	8	17	17
*F. fujikuroi* complex (n=33)	A-Se NPs	3	1	2	7	5	9	5		1		
	C-Se NPs	1	1	1	4	5	9	4	5	3		
	P-Se NPs	9	2	6	4	5	3	1	1	2		
	V-Se NPs	16	7	6	4							
	H-Se NPs			1	5	8	4	8	3	3	1	
	VRC							10	3	15	5	
	ITC										6	27
	AMB				1	1	3	11	7	6	1	3
	NAT				1	1	1	7	6	9	8	
	CAS									2	31	
	POS					2	2	2	4	7	8	8
*F. oxysporum* complex (n=7)	A-Se NPs				3		2	1	1			
	C-Se NPs						5	2				
	P-Se NPs				5		2					
	V-Se NPs	3	1	3								
	H-Se NPs						4	2	1			
	VRC							1	4	1	1	
	ITC											7
	AMB						1		3	2		1
	NAT								3	1	3	
	CAS										7	
	POS						2				3	2
*F. incarnatum equiseti* species complex (n=1)	A-Se NPs					1						
	C-Se NPs						1					
	P-Se NPs							1				
	V-Se NPs	1										
	H-Se NPs								1			
	VRC									1		
	ITC											1
	AMB							1				
	NAT										1	
	CAS										1	
	POS								1			
*F. sambucinum* species complex (n=1)	A-Se NPs				1							
	C-Se NPs	1										
	P-Se NPs				1							
	V-Se NPs	1										
	H-Se NPs	1										
	VRC									1		
	ITC											1
	AMB							1				
	NAT										1	
	CAS										1	
	POS								1			

MIC: minimum inhibitory concentration, MEC: minimum effective concentration, A-Se NPs: *A. paradoxum*-selenium nanoparticles, C-Se NPs: *C. caspius*-selenium nanoparticles, P-Se NPs: *P. vera* L. hull-selenium nanoparticles, V-Se NPs: *V. faba* L. hull-selenium nanoparticles, H-Se NPs: *H. persicum*-selenium nanoparticles, VRC: voriconazole, ITC: itraconazole, AMB: amphotericin B, NAT: natamycin, CAS: caspofungin, POS: posaconazole

 The obtained results were extremely intriguing since all five types of biosynthesized Se NPs demonstrated significantly higher antifungal activity, compared to routine antifungal drugs in the clinical settings.

According to GM MICs/MECs values, V-Se NPs (0.03 μg/ml) showed the lowest GM MICs/MECs values, followed by P-Se NPs (0.25 μg/ml), A-Se NPs (0.39 μg/ml), C-Se NPs (0.55 μg/ml), H-Se NPs (0.9 μg/ml), amphotericin B (1.05 μg/ml), voriconazole (3.25 μg/ml), natamycin (3.91 μg/ml), posaconazole (5.29 μg/ml), caspofungin (7 μg/ml), and itraconazole (13.01 μg/ml). Moreover, the comparison of the susceptibility results revealed that the *Fusarium* strains had the lowest MIC90/MEC90 values for V- Se NPs (0.125 μg/ml), followed by A-Se NPs (2 μg/ml), P-Se NPs (4 μg/ml), C-Se NPs (4 μg/ml), H-Se NPs (4 μg/ml), amphotericin B (4μg/ml), voriconazole (8 μg/ml), natamycin (8 μg/ml), caspofungin (8 μg/ml), posaconazole (16 μg/ml), and itraconazole (16 μg/ml).

## Discussion

*Fusarium* is a globally distributed multidrug-resistant genus that has the potential to cause a wide range of infections in humans [ 19 ]. In the present study, the inhibitory activity of five plant-based biosynthesized Se NPs and six common antifungal drugs were tested against 98 clinical *Fusarium* strains. Remarkably, it was discovered that the antifungal activity of plant-based Se NPs was superior to that of voriconazole, itraconazole, amphotericin B, posaconazole, natamycin, and caspofungin *in vitro*.

The lowest GM MICs and the highest antifungal activity among the biosynthesized selenium nanoparticles belonged to V-S eNPs (0.03 μg/ml), followed by P-Se NPs (0.25 μg/ml), A-Se NPs (0.39 μg/ml), C-Se NPs (0.55 μg/ml), and H-Se NPs (0.9 μg/ml). Additionally, the MIC_50_ values of all five types of biosynthesized Se NPs against *Fusarium* isolates were significantly lower than those of the drugs of choice for the treatment of invasive fusariosis, which were amphotericin B (MIC_50_, 1 μg/ml) and voriconazole (MIC_50_, 4 μg/ml). 

Selenium has antimicrobial properties and has been shown to inhibit the growth of fungi and bacteria. Selenium derivatives, such as selenium sulphide, are commonly used in the treatment of pityriasis versicolor [ 36 - 40 ]. Nanotechnology advancements in recent years have provided a safe strategy for the reduction of selenium toxicity. When compared to inorganic and organic forms, Se NPs have lower toxicity and higher bioavailability [ 24 , 41 - 43 ]. 

Several studies have shown that biogenic selenium nanoparticles have antifungal activity [ 29 , 44 , 45 ]. Shakibaie et al. investigated the antifungal activity of *Bacillus* species synthesized selenium nanoparticles against *A. fumigatus* and *Candida albicans*, and the measured MICs for *C. albicans* (70 μg/ml) and *A. fumigatus* (100 μg/ml) showed that the biogenic Se NPs had good antifungal activity [ 44 ]. Shahverdi et al. also investigated the antifungal activity of *Klebsiella pneumoniae*-produced selenium nanoparticles against clinical isolates of the *Malassezia* and *Aspergillus* genus. The MICs for all fungal strains were within the range of 10-260 g/ml, with *M. sympodialis*, *M. furfur*, and *A. terreus* showing the highest antifungal activity [ 45 ]. 

Another study assessed the antifungal activity of Se NPs synthesized by *Lactobacillus acidophilus* in controlling wheat crown and root rot diseases caused by *Fusarium* species, and biogenic Se NPs successfully inhibited fungal growth at concentrations ranging from 20 to 40 μg/ml [ 46 ]. Furthermore, several studies on the synthesis of nanoparticles using plant extracts have been published [ 47 - 49 ]. 

There have been few studies on the antifungal activity of biosynthesized Se NPs derived from plant extracts. Gunti et al. demonstrated that biosynthesized Se NPs derived from *Emblica officinalis* fruit extract had strong antifungal activity. The MIC values were found to be between 07.50 ± 1.32 and 25.50 ± 2.78 μg/ml. The lowest and highest found MIC values were 07.50 ± 1.32 μg/ml against *Rhizopus stolonifer* and 25.50 ± 2.78 μg/ml against *A. oryzae*, respectively [ 50 ]. 

In another study, Kokila et al. biosynthesized Se NPs from *Diospyros montana* extract and reported antimicrobial activity in the form of zone of inhibition values of 08, 07, and 08 mm against *Staphylococcus aureus*, *Escherichia coli*, and *A. niger*, respectively [ 51 ]. Ali et al. also investigated the antifungal activity of Se NPs extracted from *Capparis decidua* fruit against *C. albicans* and discovered that biosynthesized Se NPs have a high antifungal activity [ 52 ]. 

In the present study, all five biosynthesized Se NPs derived from plant extracts outperformed the commonly used antifungal drugs. Biosynthesized Se NPs, particularly nanoparticles made from *V. faba L*. hull, *P. vera L*. hull, and *A. paradoxum* plant extracts demonstrated potent antifungal activity against clinical *Fusarium* isolates *in vitro*. In addition to the antifungal properties of metal ions and the size of the nanoparticles, the antifungal activity of Se NPs *in vitro* may be attributed to the phytochemical composition of the plant extracts used for green synthesis, as these factors may affect their bioavailability and antimicrobial activity [ 30 , 32 ]. 

## Conclusion

Plant-based Se NPs demonstrated supreme antifungal activity and could be considered promising antifungal agents for Fusarium infections. However, tests, such as toxicity and *in vivo* tests, are needed before the product can be used in clinical settings.
